# Sphingosine-1-phosphate as an indicator for deciding the use of adjuvant corticosteroids therapy in community-acquired pneumonia (sphingosine-1-phosphate and pneumonia trial): Study protocol for a randomized, placebo-controlled trial

**DOI:** 10.1097/MD.0000000000017278

**Published:** 2019-09-20

**Authors:** Shih-Chang Hsu, Wen-Cheng Huang, Chung-Te Liu, Yuan-Pin Hsu, Jer-Hwa Chang, Shau-Ku Huang, Chin-Wang Hsu

**Affiliations:** a Emergency Department, Department of Emergency and Critical Medicine, Wan Fang Hospital; b Department of Emergency Medicine, School of Medicine; c Graduate Institute of Clinical Medicine, College of Medicine; d Division of Nephrology, Department of Internal Medicine, Wan Fang Hospital; e Department of Internal Medicine; f Division of Pulmonary Medicine, Department of Internal Medicine, School of Medicine, College of Medicine, Taipei Medical University; g Division of Pulmonary Medicine, Department of Internal Medicine, Wan Fang Hospital, Taipei Medical University, Taipei; h Research Center for Environmental Medicine, Kaohsiung Medical University, Kaohsiung; i National Institute of Environmental Health Sciences, National Health Research Institutes, Miaoli County, Taiwan; j Lou-Hu Hospital, Shen-Zhen University, Shen-Zhen, China; k Johns Hopkins Asthma and Allergy Center, Johns Hopkins University School of Medicine, Baltimore.

**Keywords:** adjuvant corticosteroids therapy, community-acquired pneumonia, sphingosine-1-phosphate

## Abstract

**Introduction:**

Pneumonia is one of the leading causes of death worldwide, represents a potentially life-threatening condition. In recent studies, adjuvant corticosteroids therapy has been shown to improve outcome in severe community-acquired pneumonia (CAP); however, the treatment response to corticosteroids vary. It is important to select patients likely to benefit from the treatment. Currently, the optimal patient selection of corticosteroids treatment is not yet clearly defined.

**Methods:**

Sphingosine-1-phosphate and pneumonia (SOPN) trial is a double-blinded, randomized, placebo-controlled trial that will investigate if sphingosine-1-phosphate (S1P) can be an indicator for initiating adjuvant corticosteroids therapy in patients with severe CAP. Participants will be recruited from the emergency department and randomized to receive 20 mg of methylprednisolone twice daily or placebo for 5 days. The primary outcome will be “in-hospital mortality.” Secondary outcomes will include intensive care unit (ICU) admission, length of ICU stay, length of hospital stay, and clinical outcomes at Day 7 and Day 14.

**Conclusion:**

SOPN trial is the first randomized placebo-controlled trial to investigate whether S1P can be a predictive biomarker for adjuvant corticosteroids therapy in patients with severe CAP. The trial will add additional data for the appropriate use of adjuvant corticosteroids therapy in patients with severe CAP. Results from this clinical trial will provide foundational information supporting that if the S1P is appropriate for guiding the patient selection for corticosteroids adjuvant therapy.

## 1 Introduction

Pneumonia is the most frequent infectious cause of death worldwide^[1]^ and imposes a considerable burden on healthcare resources. Despite the advancement in treatment and diagnosis, the overall 30-day mortality rate of community-acquired pneumonia (CAP) is as high as 12.1% for patients who were 65 years of age or older and admitted to hospital; moreover, for a patient with severe CAP, the mortality rate could reach 27%.^[2,3]^ Recently, a few meta-analysis results suggested that the use of corticosteroids adjunctive therapy outweighs possible harms in patients with CAP, who are at high risk of mortality.^[4–6]^ Those studies demonstrated that corticosteroids reduce mortality, treatment failure, complication rates, and length of hospitalization. They further suggested that adjunctive corticosteroid therapy is associated with hyperglycemia, but the harm does not outweigh the benefits. Although patients with severe CAP could benefit from adjunctive corticosteroid therapy, the optimal patient selection, dosing, and duration of corticosteroids were not yet clearly defined.

Corticosteroids inhibit the expression and function of several cytokines involved in the inflammatory response associated with pneumonia.^[7]^ Lately, the involvement of sphingosine-1-phosphate (S1P) in steroid function had been suggested.^[8]^ Also, our recent observational study showed significantly elevated S1P levels in patients who were treated with methylprednisolone during hospitalization.^[9]^ S1P is a bioactive sphingolipid with a documented regulatory role in immune defense and maintenance of endothelial barrier integrity.^[10,11]^ Therefore, we believe that S1P plays an important role in the pathophysiology of pneumonia.

Several studies have demonstrated the effects of adjunctive corticosteroids therapy on the patient with severe CAP.^[12–14]^ Since the information of patient selection, dosing, and duration of steroids is limited, the clinical use of adjunctive corticosteroids therapy becomes difficult. Therefore, to identify the potential biomarker for patient selection of the therapy is essential. This double-blind, placebo-controlled, randomized controlled trial will investigate whether S1P can be a biomarker for guiding the use of corticosteroids adjuvant therapy.

## 2 Material and methods

### 2.1 Objectives

The primary objective of the sphingosine-1-phosphate and pneumonia (SOPN) trial is to determine whether S1P can be an indicator for the use of corticosteroids adjuvant therapy in patients with severe CAP.

### 2.2 Study setting

The SOPN trial is being conducted at Wan Fang Medical Center, Taipei, Taiwan. The hospital is affiliated with Taipei Medical University and is located within the metropolitan area of Taipei City, Taiwan, at Wenshan District, and has had more than 65,000 emergency visits annually. Wenshan is a district of 273,872 inhabitants according to the 2018 registry office, located in the south of Taipei City.

### 2.3 Study design

This is a prospective, double-blinded, randomized placebo-controlled trial in adults in accordance with standard protocol items: recommendations for interventional trials (SPIRIT) guidelines. We plan to enroll 400 individuals with CAP and Pneumonia Severity Index (PSI) >90. Patients are eligible if they meet the following inclusion criteria were: age ≧18 years and suspected diagnosis of CAP as defined by the Infectious Disease Society of America/American Thoracic Society Consensus Guideline and PSI >90. Patients are excluded from the study if one of the following exclusion criteria applied: presence of severe immunosuppression (human immunodeficiency virus infection, use of immunosuppressants), malignancy, pregnancy or breastfeeding, current use of antibiotics or corticosteroids, any likely infection other than CAP, or pneumonia that developed within 3 days after hospital discharge. The final diagnosis will be provided by the admitting pulmonologists.

Participants will be recruited from the emergency department (ED) of Wan Fang Hospital by clinical study registered nurse. After satisfying the inclusion/exclusion criteria and giving consent, patients are consented and admitted to Wan Fang Hospital. Patients will be randomized on a 1:1 basis to receive 20 mg of methylprednisolone twice daily or placebo for a total of 5 days. Randomization is based on a one-on-one allocation, and the allocation sequence will be computer-generated by the study coordinator. All patients and treatment providers are blinded to the allocation. Unblinding is not permissible during the trial period. The study coordinator will keep the randomization information in a secure and encrypted database.

**Figure 1:**
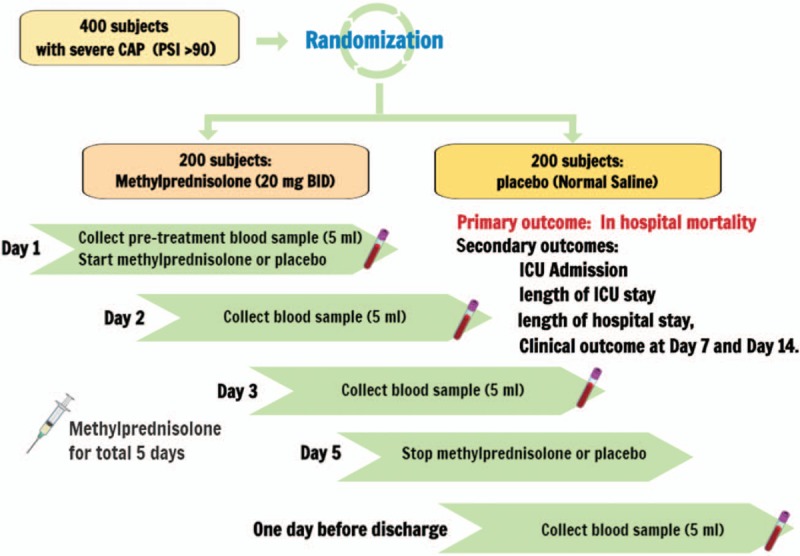
Study flow chart.

All patients are treated with antibiotics according to clinical judgment. Each recruited individual will fill out a specific questionnaire, which will include lifestyle, occupation, habits, and general dietary information. The initial peripheral blood sample will be obtained in the ED. Standard laboratory assessment will be performed on ED presentation. The blood samples will be drawn each day of hospitalization until Day 3 and 1 day before a planned discharge for S1P measurement. Figure 1 represents the study flow chart of the overview of the study design and progression of participants through the study.

The following parameters will be recorded for each participant: sex, age, body weight, body temperature, vital signs at the ED, and clinical characteristics of CAP. The laboratory testing will include baseline analyses (hematocrit, white blood count with differential, serum sodium, potassium, and chloride), Alanine Transaminase, Aspartate aminotransferase, C-Reactive Protein, Blood urea nitrogen, and creatinine. The plasma S1P will also be tested and will be measured by enzyme-linked immunosorbent assay. The questionnaire will provide the individual's basic information on personal habits and family history for further analysis.

### 2.4 Outcomes

The primary outcome is “in-hospital mortality.” Secondary outcomes included

(1) Intensive care unit (ICU) admission;
(2) Length of ICU stay;
(3) Length of hospital stay,
(4) Clinical outcomes at Day 7 and Day 14 (Table 1).

**Table 1:**
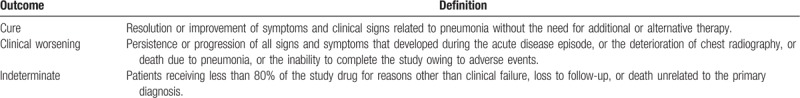
Clinical outcomes at Day 7 and Day 14.

### 2.5 Data collection and management

Outcomes will be collected by a blinded research assistant. Data will be directly entered into a secure, custom-built database at the time of data collection. The integrity of data will be closely monitored for omissions and errors. Research assistants will be trained to ensure data accuracy, consistency, and completeness. All serious adverse events and adverse events will be recorded according to the established protocol and then presented to the Ethics Committee of Taipei Medical University. The committee oversees the SOPN trial to monitor quality control of the data and safety aspects of the study. All data will be stored securely in either locked filing cabinets or electronically database with access granted only to the study coordinator and principal investigator (Chin-Wang Hsu). The principal investigator will have access to the final data set. We planned to perform interim analyses during April 2021. The principal investigator and Ethics Committee of Taipei Medical University will have access to the results and can make the final decision to terminate the trial.

### 2.6 Data analysis and sample size

Based on previous trials,^[15,16]^ we assumed a mortality rate of 15% in the placebo group and 5% in the corticosteroid group. A sample size of 200 per group (400 total) will be sufficient to detect a between-group difference, assuming a power of 90%, *α* of 5%, and allowing for 5% dropout rate. First, patients who are placed on systemic corticosteroid therapy will be compared with those not receiving corticosteroids. Then the patients will be divided into 2 groups based on the initial serum S1P level. According to our previous study,^[9]^ low S1P producer is defined as a serum S1P concentration of less than 25 ng/mL. The effects of adjuvant corticosteroid therapy will be assessed in the low S1P producer and normal S1P producer group. The optimal cut-off value of S1P concentration will also be investigated.

Statistical analysis of the data obtained in the study will be made using R 3.2.4 software (R Foundation for Statistical Computing, Vienna, Austria). The casewise deletion of missing data method will be used for handling missing data. Continuous variables are expressed as means and standard deviation or medians and the interquartile range where appropriate. The categorical variables are expressed as counts or percentages. Continuous variables were compared using *t* test or Mann–Whitney *U* test depending on data distribution. The categorized data will be assessed by using the *χ*^2^ test or Fisher direct exact test as appropriate. The degree of association between variables will be measured by the Spearman rank correlation test. The Kaplan–Meier method will be used to analyze time from admission to discharge and survival. Statistical tests will be 2-sided, and a value of *P* < .05 was considered statistically significant.

### 2.7 Trial status

At the time of manuscript submission, the trial is ongoing. The actual study start date (version 1.0) is June 15, 2019

### 2.8 Ethics and dissemination

The study will be conducted according to the Helsinki Declaration, and the Guideline for Good Clinical Practice of the International Conference of Harmonisation. This protocol is written following the SPIRIT 2013 guidelines and was approved by the Ethics Committee of Taipei Medical University – Joint Institutional Review Board (TMU-JIRB NO: N201812054). The results of this study will be submitted for publication in peer-reviewed journals.

## 3 Discussion

Patients with severe CAP would benefit from adjunctive corticosteroid therapy; however, the ideal patient selection, treatment duration, and optimal dosing of corticosteroid are largely unknown. The rationale of corticosteroids adjunctive therapy is that systemic corticosteroid could attenuate local and systemic inflammatory response and inhibit the expression of proinflammatory cytokines.^[17]^ Recently, an alternative mechanism of glucocorticoid action was proposed by a group from Germany. They suggested that increased circulating S1P levels resulting from the induction of sphingosine kinase 1 by glucocorticoids were essential for the inhibition of pulmonary inflammation.^[8]^

S1P is a bioactive sphingolipid and has both extracellular and intracellular effects in mammalian cells.^[10,11,18,19]^ S1P is involved in many physiological processes, including immune responses and endothelial barrier integrity.^[20–23]^ In the context of endothelial barrier integrity, S1P plays a crucial role in protecting the lungs from the pulmonary leak and lung injury.^[24–27]^ Because of the involvement in lung injury and endothelial barrier function, S1P may be a potential biomarker of pneumonia. Moreover, a recent study proposed that targeting the S1P/S1P receptor 2-signaling pathway in the lung may provide a novel therapeutic perspective in pneumonia for the prevention of acute lung injury.^[28]^

Recently, our study suggests that patients with CAP have significantly higher plasma S1P levels than controls. Further, the S1P levels were found to be inversely correlated with PSI score, CURB-65 (Confusion, Urea, Respiratory rate, Blood pressure and age ≥65) score, and hospital length of stay in patients with CAP. Our initial findings suggest that plasma S1P is a potential biomarker for predicting prognosis in CAP.^[9]^ In patients with CAP, the lung vascular barrier function is often compromised; therefore, higher S1P levels could be potentially beneficial in enhancing the endothelial functions. In CAP, although inflammation is important in host defense, exaggerated inflammation can damage the lungs, contributing to pulmonary dysfunction. Also, an increase in circulating S1P level could attenuate pulmonary inflammation. As a result, patients with CAP who were unable to produce a sufficient amount of S1P may have a poor prognosis.

Based on the above evidence, we hypothesized that the S1P plays a vital role in the pathobiology of pneumonia. Moreover, S1P is not only a useful biomarker for prognosis of CAP, but also can be an indicator for deciding the use of corticosteroids adjuvant therapy in patients with severe CAP. Recently, a group from Mayo Clinic had also designed a clinical trial by using C-reactive protein to guide the use of corticosteroids (SMART Trial; ClinicalTrials.gov Identifier: NCT03852537). To the best of our knowledge, this is the first study to investigate the selection of patients for adjunctive corticosteroid therapy. In this trial, we try to demonstrate that S1P is a biomarker for guiding the patient selection for corticosteroids adjuvant therapy. We also seek to find the optimal cut-off value of S1P concentration for initiating corticosteroids adjuvant therapy.

## Author contributions

**Conceptualization:** Shih-Chang Hsu, Wen-Cheng Huang, Chung-Te Liu, Yuan-Pin Hsu, Shau-Ku Huang, Chin-Wang Hsu.

**Formal analysis:** Shih-Chang Hsu, Chung-Te Liu.

**Funding acquisition:** Chin-Wang Hsu.

**Investigation:** Shih-Chang Hsu.

**Methodology:** Shih-Chang Hsu, Wen-Cheng Huang, Chung-Te Liu, Yuan-Pin Hsu, Jer-Hwa Chang.

**Project administration:** Chin-Wang Hsu.

**Resources:** Wen-Cheng Huang, Shau-Ku Huang, Chin-Wang Hsu.

**Supervision:** Jer-Hwa Chang, Shau-Ku Huang, Chin-Wang Hsu.

**Writing – original draft:** Shih-Chang Hsu, Yuan-Pin Hsu.

**Writing – review & editing:** Wen-Cheng Huang, Chung-Te Liu, Jer-Hwa Chang, Shau-Ku Huang, Chin-Wang Hsu.

Shih-Chang Hsu orcid: 0000-0003-1010-1182.
